# Bidirectional links between HIV and intimate partner violence in pregnancy: implications for prevention of mother-to-child transmission

**DOI:** 10.7448/IAS.17.1.19233

**Published:** 2014-11-03

**Authors:** Abigail M Hatcher, Nataly Woollett, Christina C Pallitto, Keneuoe Mokoatle, Heidi Stöckl, Catherine MacPhail, Sinead Delany-Moretlwe, Claudia García-Moreno

**Affiliations:** 1 Wits Reproductive Health and HIV Institute, University of the Witwatersrand, Johannesburg, South Africa; 2 Division of HIV/AIDS, University of California, San Francisco, CA, USA; 3 Department of Reproductive Health and Research, World Health Organization, Geneva, Switzerland; 4 Department of Global Health and Development, London School of Hygiene and Tropical Medicine, London, UK; 5 Collaborative Research Network for Mental Health and Wellbeing, University of New England, New South Wales, Australia

**Keywords:** HIV, prevention of mother-to-child transmission, intimate partner violence, pregnancy, adherence

## Abstract

**Introduction:**

Prevention of mother-to-child transmission (PMTCT) has the potential to eliminate new HIV infections among infants. Yet in many parts of sub-Saharan Africa, PMTCT coverage remains low, leading to unacceptably high rates of morbidity among mothers and new infections among infants. Intimate partner violence (IPV) may be a structural driver of poor PMTCT uptake, but has received little attention in the literature to date.

**Methods:**

We conducted qualitative research in three Johannesburg antenatal clinics to understand the links between IPV and HIV-related health of pregnant women. We held focus group discussions with pregnant women (*n*=13) alongside qualitative interviews with health care providers (*n*=10), district health managers (*n*=10) and pregnant abused women (*n*=5). Data were analysed in Nvivo10 using a team-based approach to thematic coding.

**Findings:**

We found qualitative evidence of strong bidirectional links between IPV and HIV among pregnant women. HIV diagnosis during pregnancy, and subsequent partner disclosure, were noted as a common trigger of IPV. Disclosure leads to violence because it causes relationship conflict, usually related to perceived infidelity and the notion that women are “bringing” the disease into the relationship. IPV worsened HIV-related health through poor PMTCT adherence, since taking medication or accessing health services might unintentionally alert male partners of the women's HIV status. IPV also impacted on HIV-related health via mental health, as women described feeling depressed and anxious due to the violence. IPV led to secondary HIV risk as women experienced forced sex, often with little power to negotiate condom use. Pregnant women described staying silent about condom negotiation in order to stay physically safe during pregnancy.

**Conclusions:**

IPV is a crucial issue in the lives of pregnant women and has bidirectional links with HIV-related health. IPV may worsen access to PMTCT and secondary prevention behaviours, thereby posing a risk of secondary transmission. IPV should be urgently addressed in antenatal care settings to improve uptake of PMTCT and ensure that goals of maternal and child health are met in sub-Saharan African settings.

## Introduction

Prevention of mother-to-child transmission (PMTCT) has reduced new infant HIV infections from an estimated 32% in the absence of treatment [1, 2], to as low as 1% [3, 4]. However, major gaps in achieving PMTCT coverage remain. In 21 priority African countries, PMTCT coverage is estimated to be 65% [5]. A recent meta-analysis in low- and middle-income settings suggests that while 75% of pregnant women adhere to antiretroviral therapy (ART) during pregnancy, only 53% maintain adequate adherence levels in the postpartum period [6]. Ensuring PMTCT adherence is crucial, particularly as countries increasingly move towards “Option B+,” a policy that offers immediate, lifelong treatment for pregnant women living with HIV [7].

Many structural drivers influence PMTCT uptake and adherence. The literature has noted that structural factors such as stigma [8–13], poverty [11] and transport costs [10] inhibit women's ability to adhere to PMTCT. Another key structural factor shaping access and adherence to PMTCT may be intimate partner violence (IPV). Fear and experience of IPV influence pregnant women's decisions to take up HIV services [14, 15], and anticipated violence is associated with declines in HIV testing among pregnant women [12, 16–22]. A history of physical or sexual violence decreases the likelihood of HIV-positive women using ART when medically eligible [23, 24], and those who experience abuse are more likely to miss clinic visits and delay linkage to care [25].

Little research to date has explored the association between IPV and PMTCT. In one qualitative study in South Africa, IPV was described as a common barrier to ART adherence in pregnancy [11]. Healthy intimate partner relationships improve PMTCT uptake: male involvement in antenatal care predicted better adherence to nevirapine in one South African study [26]; male antenatal attendance halved the risk of MTCT in a Kenyan study, an association that persisted after controlling for maternal viral loads [27].

Using qualitative research methodology, we explored IPV as a potential structural driver of HIV-related health among pregnant women. This research aimed to contribute to literature suggesting that structural drivers shape the health and well-being of those already living with HIV, and may pose barriers to uptake of proven prevention strategies.

## Methods

We conducted qualitative research to explore the links between IPV and HIV-related health among pregnant women and service providers in Johannesburg, South Africa. This research was a portion of a larger formative study, intended to help our team design an intervention to address IPV in pregnancy. In this setting, an estimated 29% of pregnant women are HIV positive [28] and between 25 and 35% experience physical or sexual IPV in the 12 months leading up to pregnancy [29–32].

### Conceptual framework

**Figure 1:**
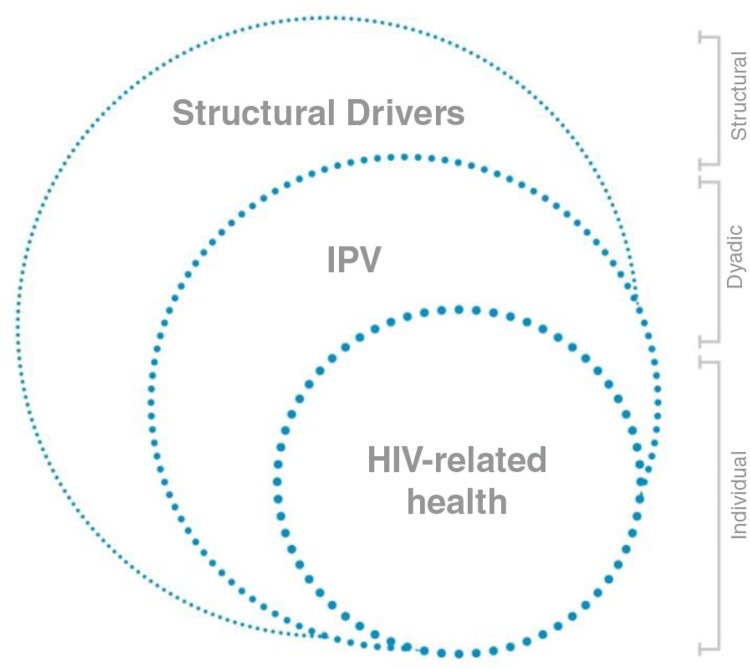
Conceptual framework.

To explore the relationship between IPV and HIV-related health of pregnant women, we used an adapted socio-ecological conceptual framework (Figure 1), which posits that broader structural factors and relationship characteristics influence a woman's HIV-related health [33]. This type of approach has been embraced by social scientists, who note that broader social and societal factors shape how women are able to adhere to ART [34] and the extent to which they experience IPV [35]. A socio-ecological framework highlights that the structural context influences the conditions and health outcomes of both IPV and HIV.

### Data collection

We conducted an exploratory qualitative study using in-depth interviews (IDIs) and focus group discussions (FGDs) with a wide range of stakeholders with the potential to take part in, deliver, or scale-up an intervention for violence in pregnancy. Participants included pregnant women, pregnant women experiencing IPV, health workers, non-governmental organizations, community leaders and policy makers (Table 1).

**Table 1:** Data collection methods

Participant group	Group size	Method	Sampling	Example participants	Topics
Pregnant women at ANC	*n*=13 women in 4 FGDs	Focus group discussions	Convenience	–	Social and structural drivers of IPV; types of IPV in pregnancy; patterns of help seeking and available community resources for violence and HIV; barriers to disclosing IPV; receptivity to an antenatal intervention
Pregnant abused women	*n*=5	Semi-structured interviews	Convenience	–	Existing needs and concerns of abused women; patterns of help seeking and available community resources for violence; links between IPV and HIV; receptivity to an antenatal intervention
Policy makers	*n*=10	Semi-structured interviews	Purposive	Department of Health managers, academic experts	Types of IPV in pregnancy; current health sector response to IPV; potential integration with HIV activities, including PMTCT
Health providers	*n*=8	Semi-structured interviews	Purposive	Doctors, nurses, lay counsellors in antenatal clinics	Types of IPV in pregnancy; knowledge and practice responding to IPV; receptivity of health workers to antenatal intervention; existing capacity in clinic
Non-governmental organizations	*n*=6	Semi-structured interviews	Purposive	Shelters, police, counselling services	Psycho-social, legal and other needs of abused women; referral options for women living with IPV
Community leaders	*n*=4	Semi-structured interviews	Convenience	Pastors, neighbourhood representatives, traditional healer	Community factors that support or prevent women from seeking IPV assistance during pregnancy

*Pregnant women seeking antenatal care* from two antenatal clinics were recruited for four FGDs (a total of *n*=13 women participated). Women were given group information about the study while they waited in queue for a clinic appointment. All FGDs were conducted in a private room in the clinic, led by trained moderators who were the same sex as participants and fluent in multiple local languages (Sotho, Zulu, Tswana). Semi-structured discussion guides explored several topics (Table 1). Discussions were audio-recorded after obtaining participants’ permission and signing an informed consent form. The discussion groups lasted for about 1 hour and 30 minutes, and women were reimbursed R50 (US $6). Because of the nature of focus groups, additional confidentiality measures were implemented: during the informed consent process, researchers explained that questions about women's individual experiences of violence or HIV would not be asked, but rather the discussion would address the issue as observed in the community.

*Pregnant women who were experiencing IPV* were identified during the FGDs. Trained researchers explained that those women who had personal experience of IPV and were interested in participating in IDIs could approach the research team outside of the information giving session and privately indicate their interest in taking part in an interview. The interviews (*n*=5) took place in a private room at the clinics while the pregnant women were still waiting to be seen by clinic staff. As shown in Table 1, the topics explored through structured interview guides were more focused on IPV-related help seeking and the relationship between IPV and HIV. On average, these interviews lasted about 60 minutes.

In depth interviews with *Other Key Stakeholders* were led by the research team and covered similar topics. This group comprised policy makers (*n*=10), health workers (*n*=8), non-governmental organizations (*n*=6) and community-based organizations (*n*=4). Stakeholder interviews focused on service provision and asked questions about available resources for women experiencing IPV. Some anecdotes of cases were shared, but this was not the main rationale for these interviews.

Several steps were taken to ensure confidentiality and provide additional support for participants during the research. In keeping with ethical considerations of researching IPV in pregnancy, all researches were conducted based on the World Health Organization's guidance on ethical and safety considerations in researching violence against women [36]. Study staff were trained to describe research as the “social barriers” to use health services in the community, so as to reduce any undue risk associated with participating in a violence-related study. All participants were offered an information sheet containing contact information of local resources (counselling, legal advice and health care). Given the high prevalence of IPV in South Africa, it was likely that participants in categories other than “pregnant and experiencing IPV” category had experienced or witnessed IPV. If any individual demonstrated a need for additional assistance, that individual was offered an opportunity to speak to someone about his or her experience of IPV, and given referrals to organizations that could assist him or her. However, no participants asked for this referral during the course of the formative research.

All participation in this formative research was sought on the basis of informed consent and good clinical practice guidelines. Ethics approval was obtained by the World Health Organization (WHO A65780) and University of the Witwatersrand (HREC M110832).

### Data analysis

The interview and FGD data were transcribed verbatim in the language in which they were conducted and, as necessary, translated from the local language (Sotho, Zulu, Tswana) into English by professional translators. To ensure accurate translation, each transcript was reviewed by a researcher, and queries were resolved through discussions among the researchers via phone or email. All identifying information about the participant or clinic setting was removed and transcripts were saved by a file name with no personal details.

Data were managed in QSR Nvivo 10, a qualitative analysis software package, following a two-day qualitative management and analysis training of the research team. Members of the research team collaboratively built an analytical framework of broad codes by creating a “start list” of possible themes and building upon the research questions. Each broad code, or wide thematic basket of ideas [37], was applied to each transcript by two researchers using NVivo. The research team then held a series of meetings to collectively develop “fine codes” using an inductive approach – deriving meaning from the data itself rather than imposing pre-formed ideas [38]. Fine codes were developed by printing out a full set of excerpts (from each database) related to each code and identifying sub-themes emerging from the data.

## Results

**Figure 2:**
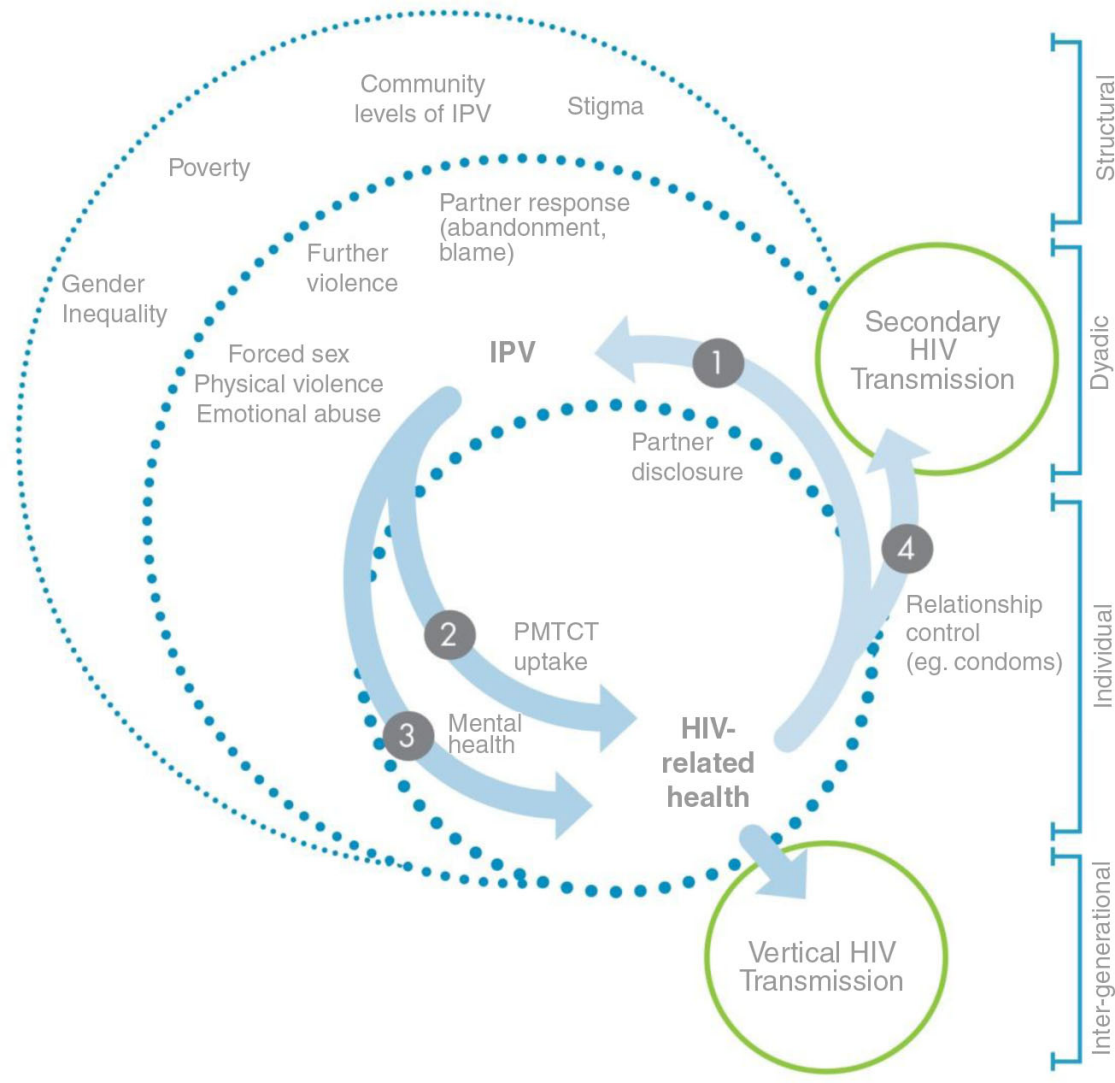
IPV and HIV-related health among pregnant women.

We found qualitative evidence of strong bidirectional links between IPV and HIV among pregnant women. Here, we present a conceptual framework (Figure 2) for understanding the ways in which IPV is related to HIV-related health of pregnant women.

### Pathway 1: HIV diagnosis leads to IPV via partner disclosure

HIV diagnosis during pregnancy was noted to be a trigger of IPV. One pregnant woman described how severe violence began following disclosure of her HIV-positive status during pregnancy:

He started telling me things, hurting me emotionally, telling me that I'm a fool, and stupid, I'm an idiot. And then he strangled me, That's when it started … Maybe it's pregnancy, I don't know. I told him that I am HIV positive, so I don't know if that's what made him to do all these things. – Pregnant abused woman 1

HIV may lead to violence because it causes relationship conflict during the disclosure process. Usually, the conflict is related to perceived infidelity and blaming women for “bringing” the disease into the relationship:

Yes, if you're HIV positive, you start blaming each other. Because maybe the husband will be saying the wife brought it. So sometime, there's a connection [between HIV and violence] because you end up blaming each other. – Pregnant woman, FGD 3

Because HIV testing is coupled with antenatal care, women often learn of their HIV status in a clinic during access to health care during pregnancy. Within this health care context, women bear the brunt of disclosure to partners, who tend to use women's status as a “proxy” for their own.

In addition to physical violence, pregnant women described experiencing emotional abuse and abandonment following disclosure of HIV to a partner:

I have a sister, she was pregnant, … then she came to be tested. When she tested she found out she is positive, and when she told her boyfriend everything turned around. And there was violence at home. He started coming late and when she started asking for things for her and the baby, he started to react badly up until he ended up leaving her. – Pregnant woman, FGD 2

Within a context where women fear violence, blame and abandonment, it is perhaps not surprising that many pregnant women chose not to disclose their HIV status to partners. Several pregnant women spoke about the fear of partner disclosure when women live in violent relationships:

Women who are in this abusive relationship, they do get HIV and they are scared what their partner will say. – Pregnant abused woman 1

Health workers talked about how women in violent relationships would be hesitant to disclose their status to partners:

When you counsel them … after they have tested positive and when you have to issue the treatment she'll be saying, ‘I am not going to disclose. I mustn't take this, I must hide it’. Then you find out is it a problem for her to disclose because there's some emotional abuse or physical abuse from the partner. – Female Health Worker 4

Thus, fear of partner disclosure may be an early warning sign that pregnant women are in violent or unsupportive relationships and require additional assistance during antenatal care.

### Pathway 2: IPV worsens HIV-related health via non-adherence

For women in violent relationships, adherence to PMTCT services was challenging, since taking medication or accessing health services might unintentionally alert male partners of their HIV status.

Health workers noted that non-adherence also served as warning sign that HIV-positive patients were in a violent relationship:

It was the very same patient that you had told she was HIV positive that was scared to go and disclose to their partner. It is the very same patient that will default on their medication because their partner does not know that they are taking the medication. – Policy Maker 9

In the antenatal clinic, an HIV diagnosis in the context of living with violence may cause patients to default on clinic visits:

But in your normal facility it is a bit difficult to avoid losing patients. I think we do. Especially the mere fact that you say to a patient, “you are HIV positive.” And this is a patient who is facing domestic violence! Some will just disappear. – Policy maker 3

Thus, the fear of being identified by a male partner as being HIV positive may preclude women from returning to clinic services that are essential for their health. While no participants mentioned this directly, it is important to note that non-adherence to PMTCT regimens greatly increases the risk that pregnant or breastfeeding women will transmit HIV to the infant.

### Pathway 3: IPV worsens HIV-related health via mental health

Declines in mental health were noted in women experiencing IPV in pregnancy. In response to persistent violent relationships, women described internalizing the abuse and assuming that they had done something wrong to deserve it:

He used to beat her while she was pregnant. She just accepted it, and sometime she'd blame herself. Saying maybe I'm the one who's wrong that's why he's beating me. – Pregnant woman, FGD 1

Although IPV is associated with common mental health disorders in pregnancy, few patients or providers recognized these as having clinical implications. Most health providers equated mental health to severe cases of psychopathology and said they rarely encountered mental health disorders. For example, one health worker only considered mental health in relation to bipolar depression and pharmacologically treated patients:

Mental health, yes, I remember we've had three that we already on treatment, and will tell you, I have a bipolar patient. – Health worker 8

We found that health workers often fail to notice the mental health dynamics of IPV in pregnancy, choosing instead to focus on physical health sequelae of pregnancy. For example, one health provider was asked about stress related to IPV, but responded only in terms of how stress impacts hypertension while ignoring the relevant impact on a woman's mental health:

Stress is one of the predisposing factors to the development of hypertension. So it is still there, this stress, but as a predisposing factor. Sometimes because of the pregnancy itself, you can develop hypertension of pregnancy. – Health worker 6

This tendency towards equating mental health with severe illness may be related to the lack of capacity within South Africa's public health system. As one policy maker explained, in overlooking mental health issues, current health systems make it unlikely that patients will receive the crucial support that they require:

No one has time for mental health because there are so many other crises in the health system that need to be addressed, that are much more manifested. So that means that things like depressive disorder or mental health disorders, they're not addressed – including partner depression, mental health and abuse and all of that. And people are not really encouraged to go and get support that they require. – Policy Maker 9

The notion of overlooking mental health is illustrated in an interview with one pregnant, abused woman when she described severe physical violence as leading to a state of being “a little depressed”:

Interviewer (I): Are you enjoying your pregnancy so far?

Participant (P): Being honest, a little depressed but I'm enjoying it.

I: Ok, so the depression is from what, if I may ask?

P: From the father of the baby. We are having problems.

I: What did he do, if you don't mind telling me.

P: He strangled me and then he let his cousin beat me up. – Pregnant abused woman 1

Not everyone in our sample ignored the impact of mental health on a woman's health and wellbeing. For example, poor mental health had concomitant effects on physical health for one HIV-positive participant, who described “going low” emotionally because of violence, and thereafter feeling worse physically:

I'm HIV positive and I'm in this domestic violence. And if you are HIV positive and then you have a partner who is abusing you emotionally … or physically hits you, people can't talk. Maybe you can go low, maybe you can go sick. – Pregnant abused woman 3

### Pathway 4: IPV leads to secondary HIV risk via lack of relationship control

IPV led to secondary HIV risk when women were in relationships with forced sex or without power to negotiate condom use.

When we are in relationships where our partner is abusive, sometimes we can't even negotiate things like using the condom. Let's say, for instance, you know that your partner is the kind of person that has other girlfriends, but because he uses power over you, you can't negotiate those things. – Pregnant woman, FGD 4

Male partners used their control over the relationship to dictate the terms and timing of sexual activity. In one instance, a FGD revealed a story about a newly diagnosed HIV-positive woman whose partner insisted that she have sex without condoms:

There's a friend of mine that was tested alone and she had a lot of problems. The man said, I'm not HIV positive, so I'm not going to test. So the man forced her to sleep with him without a condom. And that man said ‘No! Why? We've been sleeping without a condom, but because today you went to the clinic, you're telling me we've to use a condom?’ – Pregnant woman, FGD 1

Pregnant women described balancing risks to physical safety (absence of physical harm to themselves or foetus) with health risks (of onwards HIV transmission to partners). They described making compromises between protecting themselves and the foetus and protecting themselves and partners from sexually transmitted infections:

If you are not compromising at all and you start saying “let's use condom,” he'll start having questions. Some things are better left unsaid, just for the safety part of it. – Pregnant woman, FGD 2

Many preferred staying silent on condom negotiation, in order to stay physically safe during pregnancy.

## Discussion

We found that IPV and HIV-related health were connected concerns in the lives of pregnant women in Johannesburg. IPV and HIV seemed to have distinct pathways linking them to one another within the context of pregnancy. The initial HIV disclosure could serve as a trigger for violence in pregnancy. IPV, in turn, worsened HIV-related health through key pathways of lack of adherence and poor mental health. Finally, the experience of IPV led to secondary transmission risk behaviours – both in terms of vertical transmission due to PMTCT non-adherence or secondary transmission due to risky sex.

According to our participants, IPV shapes HIV-related health outcomes among pregnant women primarily because it leads to non-adherence. While the effect of IPV on adherence has been confirmed in small studies in the United States [39–43], this association is yet to be explored among pregnant women. Pregnant and postpartum women are a crucial population within which to understand IPV and adherence, since non-adherence leads not only to morbidity and mortality of the woman but also to risk of onwards HIV transmission to her infant [3, 4]. Antenatal care provides a crucial moment to enable adherence, since a pregnant woman accesses the health system routinely and this is when many are first diagnosed with HIV.

Poor adherence among pregnant women may relate to challenges around partner disclosure [44]. In a recent systematic review of PMTCT, partner disclosure was associated with poor PMTCT uptake in a majority of both quantitative (6 of 9) and qualitative (17 of 24) studies [45]. We found that partner disclosure following HIV diagnosis in pregnancy led to enacted or feared violence. This aligns with extant literature, which suggests that fear of new or continued IPV may lead women to avoid disclosure of their status to male partners [11]. In one Nigerian study among HIV-positive pregnant women, the prevalence of IPV was 17% before HIV testing and increased to 63% after testing for HIV and disclosing status [46]. A Zimbabwean study showed that the risk of IPV in pregnancy was greatest among those women testing positive for HIV in antenatal care [47]. Non-disclosure among pregnant women is a health risk in its own right, since it poses a risk for sexual transmission of HIV if the male partner is still HIV negative [48–51] and may have an impact on the implementation of PMTCT [52].

A related but distinct pathway linking IPV to PMTCT uptake may be mental health. A growing body of literature shows that IPV leads to depression and anxiety among pregnant women [29, 53, 54], yet this link has been largely unexplored in sub-Saharan Africa in HIV-positive populations. Our findings reflect those of a qualitative study in Zambia, in which IPV, mental health and HIV are closely related in the experience of women [55]. Such interrelated “syndemic” issues [56] should be explored in future sub-Saharan African studies.

Existing research shows poor mental health has significant impact on ART adherence [57–60] and among pregnant women depressive symptoms are associated with HIV disease progression and mortality [61]. It is possible that IPV is one condition exacerbating the relationship between mental health and HIV outcomes. Indeed, one new study suggests that the link between mental health and ART adherence may be partly driven by partner conflict [62]. Despite high rates of common mental health disorders in antenatal care [63], little screening or treatment exists in South Africa [64]. Mental health will be crucial to address among HIV-positive pregnant women because of its strong relationship to IPV and its association with the uptake of PMTCT regimens [65].

Finally, IPV may worsen secondary prevention behaviours in pregnancy. Non-adherence to PMTCT regimens greatly increases the risk that pregnant or breastfeeding women will transmit HIV to the infant [66], potentially in drug-resistant form [67]. High viral loads related to non-adherence also increase the likelihood of secondary transmission to partners, particularly in the context of unsafe sex. Our research reflects existing knowledge by suggesting that IPV inhibits women's ability to negotiate condoms [68]. These findings explore such dynamics within the context of pregnancy, thereby suggesting a dual risk of mother-to-child infection and secondary transmission risk to a partner.

Our findings echo calls for addressing IPV in pregnancy [69]. Scholars note that antenatal care provides an important “window of opportunity” for women who are regularly accessing the health system [70]. Although universal screening is not recommended in settings with limited referral options and overstretched providers [71], some type of IPV assessment, provider training and targeted response may be appropriate for South African antenatal care. Indeed, a comprehensive health response to IPV will likely require either screening or case-finding – both methods that may be acceptable in South African clinics [72, 73].

### Limitations

The findings of this formative research should be examined in light of several limitations. Firstly, this study is exploratory in nature, resulting in small sample sizes of each participant group. While analysis suggested that we began to reach saturation through FGDs with pregnant women, the IDIs with pregnant women experiencing IPV were not sufficient to reach thematic saturation [74]. Secondly, the socio-ecological perspective was brought to the data analysis process after data collection. Ideally, this conceptual approach would have informed the entire data collection process, rather than simply guiding the final interpretation of findings. However, since this was a preliminary, exploratory study, it was designed to explore several intersecting issues and we applied the conceptual framework during data analysis. Finally, some of the findings may be applicable for any woman experiencing IPV, and do not necessarily highlight the specific context of pregnancy. Further research should explore the perinatal time-period in detail to determine whether the link between IPV and HIV is somehow distinct during this life stage.

## Conclusions

IPV in pregnancy leads to declines in the physical and mental health of pregnant women. Our findings underscore the negative effects of IPV as a health issue in its own right and as a barrier to PMTCT. The connection between IPV and HIV medication adherence among pregnant women has yet to be explored quantitatively in sub-Saharan Africa. In future studies, it would be ideal to find systematic methods for recruiting more robust numbers of pregnant women who experience IPV and who are living with HIV. In the parent study [75], we anticipate that by training health providers to ask about IPV confidentially and skilfully, it may be increasingly possible to reach this crucial population.

Beyond its marked impact on physical and mental health of women, IPV in pregnancy may have important implications for Option B+, as current cost-effectiveness models assume that women are willing and able to achieve 100% adherence [76]. If Option B+ is to be adopted more broadly, the effect of IPV on adherence and mental health should be carefully considered. Addressing the inter-related issues of violence and HIV will be crucial to ensure that goals of maternal and child health are met in the sub-Saharan African region.
